# Obesogenic diet in pregnancy disrupts placental iron handling and ferroptosis and stress signalling in association with fetal growth alterations

**DOI:** 10.1007/s00018-024-05192-5

**Published:** 2024-03-25

**Authors:** Jonas Zaugg, Jorge Lopez-Tello, Barbara Musial, Owen R. Vaughan, Abigail L. Fowden, Christiane Albrecht, Amanda N. Sferruzzi-Perri

**Affiliations:** 1 Department of Physiology, Development and Neuroscience, University of Cambridge, Downing Street, Cambridge, CB2 3EG UK; 2 Institute of Biochemistry and Molecular Medicine, University of Bern, Bühlstrasse 28, CH-3012 Bern, Switzerland; 3 Swiss National Centre of Competence in Research (NCCR) TransCure, University of Bern, Bern, Switzerland

**Keywords:** Placenta, Iron, Metabolism, Diabetes, Pregnancy, Obesity

## Abstract

Obesity and gestational diabetes (GDM) impact fetal growth during pregnancy. Iron is an essential micronutrient needed for energy-intense feto-placental development, but if mis-handled can lead to oxidative stress and ferroptosis (iron-dependent cell death). In a mouse model showing maternal obesity and glucose intolerance, we investigated the association of materno-fetal iron handling and placental ferroptosis, oxidative damage and stress signalling activation with fetal growth. Female mice were fed a standard chow or high fat, high sugar (HFHS) diet during pregnancy and outcomes were measured at day (d)16 or d19 of pregnancy. In HFHS-fed mice, maternal hepcidin was reduced and iron status maintained (tissue iron levels) at both d16 and d19. However, fetal weight, placental iron transfer capacity, iron deposition, TFR1 expression and ERK2-mediated signalling were reduced and oxidative damage-related lipofuscin accumulation in the placenta was increased in HFHS-fed mice. At d19, whilst TFR1 remained decreased, fetal weight was normal and placental weight, iron content and iron transporter genes (*Dmt1*, *Zip14*, and *Fpn1*) were reduced in HFHS-fed mice*.* Furthermore, there was stress kinase activation (increased phosphorylated p38MAPK, total ERK and JNK) in the placenta from HFHS-fed mice at d19. In summary, a maternal HFHS diet during pregnancy impacts fetal growth trajectory in association with changes in placental iron handling, ferroptosis and stress signalling. Downregulation of placental iron transporters in HFHS mice may protect the fetus from excessive oxidative iron. These findings suggest a role for alterations in placental iron homeostasis in determining perinatal outcomes of pregnancies associated with GDM and/or maternal obesity.

**Graphical Abstract:**

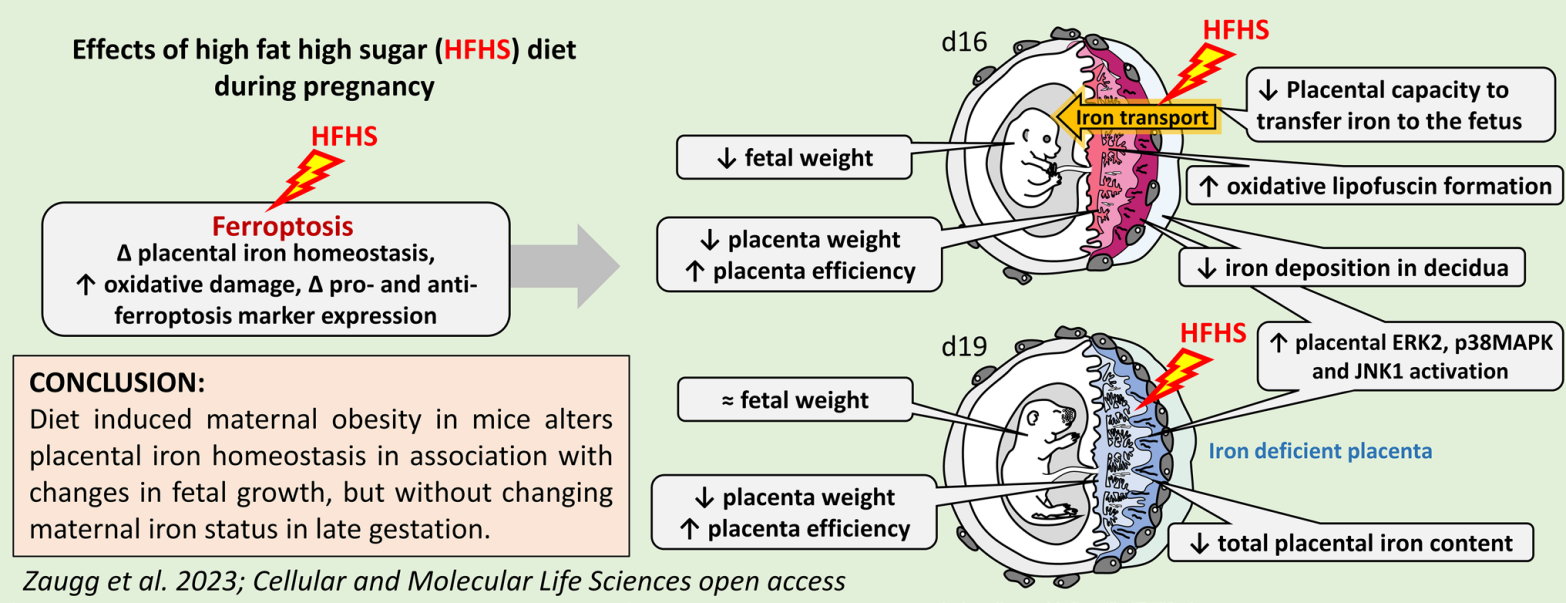

## Introduction

Throughout the last two decades, the prevalence of gestational obesity has increased tremendously. A British National Maternity and Perinatal Audit conducted between 2015 and 2016 found that more than half of the observed pregnant women (52.7%) had a body mass index (BMI) above 25, with 21.3% registered with a BMI of 30 or more [1]. Obesity is primarily caused by increased consumption of carbohydrate- and fat-rich diets that typically contain low levels of protein and micronutrients, such as essential vitamins and iron [2]. Maternal obesity increases the risk of hyperglycaemia and glucose intolerance during pregnancy, which is defined as gestational diabetes mellitus (GDM) [3–5]. Obesity and GDM also increase the risk of complications for the developing fetus and newborn, such as macrosomia, excess adiposity, preterm birth, birth injury, neonatal hypoglycaemia, and respiratory distress [6–9]. Other studies focussing on the long-term effects of obesity and GDM for the child have also found a reduction of insulin sensitivity and an increased risk to develop metabolic diseases in adulthood [10–12]. Dietary changes are common in pregnant women and may be influenced by factors, such as food aversions, cravings, time needed to prepare foods, and finances [13]. Indeed, some women crave certain food types, including carbohydrate- and fat-rich diets while expecting [14]. Despite this, the impacts of consuming a high fat and high sugar diet during pregnancy on fetal and offspring outcomes are poorly defined [15].

A mechanism by which maternal metabolic diseases during pregnancy may impact the developing child could involve altered iron homeostasis [16]. Iron requirements progressively increase to meet the demand for erythropoiesis and iron-dependent enzymes during pregnancy. Maternal and fetal iron requirements are greatest in the third trimester due to high metabolic rate, rapid fetal brain development, as well as increased blood formation in anticipation of blood loss at birth [17–19]. Hence, pregnant women are particularly vulnerable to iron deficiency and in turn, related adverse pregnancy outcomes [20]. Previous work has suggested that increased adiposity in pregnant women affects iron metabolism, as indicated by elevated hepcidin levels and decreased absorption of dietary iron [21]. Furthermore, a longitudinal, multi-racial and prospective study found women with GDM had elevated levels of ferritin in the first trimester, and a decreased soluble transferrin receptor 1 (sTFR1):ferritin closer to the time of GDM diagnosis early in second trimester [22]. Moreover, another study found a strong positive association between elevated adiposity, GDM development and maternal circulating haemoglobin levels during pregnancy [23]. Finally, both maternal obesity and excessive gestational weight gain are associated with compromised neonatal iron status [24, 25]. However, further work is needed to understand how a diet that induces obesity and metabolic derangements could impact iron homeostasis during pregnancy.

Pregnancy is generally considered as a state of increased oxidative stress, and excess adiposity and hyperglycaemic conditions in GDM can augment the oxidative state [3, 26, 27]. Excess oxidative stress occurs through a disturbance in the balance between the levels of reactive oxygen species (ROS) and the availability of antioxidants, such as glutathione (GSH) [28] and catalase (CAT), an enzyme responsible for the degradation of H_2_O_2_ to water [29]. High cellular glucose levels are known to induce the over-production of superoxide by the mitochondrial electron-transport chain [30]. Moreover, under conditions of glucose excess, glucose is converted to the polyalcohol sorbitol, which depletes intracellular GSH and leads to increased ROS levels and lipid peroxidation [31]. Hyperglycaemia can also lead to the formation of advanced glycation end products, which in turn, increase inflammation and induce cellular death and further oxidative stress [32, 33]. Importantly, altered tissue iron handling, namely iron excess is also associated with the generation of oxidative stress and oxidative damage [34–36]. Ferrous iron in the labile iron pool is highly reactive and can generate ROS by the Fenton reaction that finally leads to lipid, DNA, and protein damage, as well as ferroptosis [28]. Ferroptosis is an iron-dependant apoptosis-related cell death pathway characterized by the loss of lipid peroxide repair capacity by glutathione peroxidase 4 (GPX4) and CAT [28, 29, 37]. As a result, iron uptake, storage, utilisation, and efflux need to be strictly controlled to avoid oxidative damage and ferroptosis activation. Several pro- and anti-ferroptosis genes regulate cellular ferroptosis levels. In the case of increased ROS levels, changes to iron availability in the circulation and tissue can reduce the amount of intracellular unstable iron to prevent ferroptosis [38–40].

Over the last few years, various studies have emerged suggesting there are inter-relationships between maternal metabolic diseases, maternal iron status and oxidative stress that may have implications for pregnancy outcomes [16]. Both a low-iron diet (35 mg/kg iron) and a chow diet including an iron chelator were able to rescue the reduced insulin sensitivity and β-cell function in the *ob/ob* mouse model of type-2 diabetes [41] suggesting that the placenta has the ability to protect the fetus from hepcidin-mediated iron deficiency in obese or overweight pregnant women by a compensatory upregulation of placental TFR1 [42]. Hepcidin (HAMP) expression is stimulated by high plasma iron and iron stores and acts by binding to and inactivating the sole cellular iron exporter ferroportin (FPN1), which delivers iron from iron-acquiring cells, like enterocytes to the blood circulation [43]. Small cohort studies have also found dysregulation of placental iron homeostasis genes in diabetic pregnancies complicated by fetal iron deficiency, namely observed as elevated iron regulatory protein (IRP1) and TFR1 expression by the placenta [44]. However, there are also reports of reduced placental expression of iron transporters, ferroxidases, and iron entry regulators in GDM pregnancies [45], and hyperglycaemia has been shown to decrease expression of several iron homeostasis genes and impaired iron uptake function of a placental cell line (BeWo) in vitro [45]. Finally, supplementation with an anti-oxidant (selenium) was able to rescue the compromised expression of iron homeostasis genes induced by hyperglycaemia in BeWo cells in vitro [45]. However, the impact of maternal metabolic diseases, namely obesity and altered glucose handling during pregnancy on transplacental iron transport, its potential association with placental ferroptosis and fetal development is scarcely investigated so far.

Here, we hypothesized that a diet that induces hyperglycaemia and obesity during pregnancy induce oxidative damage, ferroptotic cellular stress responses and dysregulate iron homeostasis in the placenta with consequences for fetal growth. To test this hypothesis, we utilized a well-established mouse model, in which mouse dams are fed a western-style high fat and sugar (HFHS) diet just during pregnancy to induce excess adiposity, compromised glucose tolerance and altered insulin sensitivity [46, 47]. This study design allowed us to isolate the confounding effects of pre-existing obesity and its various associated complications (e.g. pro-inflammatory state and metabolic derangements) on placental iron handling. Using this model, we examined maternal hepcidin levels in liver lysates, iron levels in placenta and maternal liver and skeletal muscle, the expression of genes that regulate iron homeostasis and ferroptosis, and the activation of pathways involved in stress signalling in the placenta. In addition, we determined levels of oxidative damage and antioxidant potential in the placenta. Parameters were measured at gestational day (d) 16, when placental size and maternal glucose intolerance are greatest, and at d19, when maximal growth of the fetus occurs (term is – d20.5) [48].

## Material and methods

### Reagents

All chemicals and reagents were purchased from Sigma Aldrich unless otherwise stated.

### The HFHS animal model

All experiments were performed under the UK Home Office Animals (Scientific Procedures) Act 1986 following ethical review by the University of Cambridge Animal Welfare and Ethical Review Board. The present manuscript is based on a retrospective analysis of a previous and larger experiment, which characterised the effect of the HFHS diet fed only during pregnancy on maternal adiposity, glucose, and insulin handling [46, 47]. Hence, this study represents a subset of the original larger cohort.

**Fig. 1:**
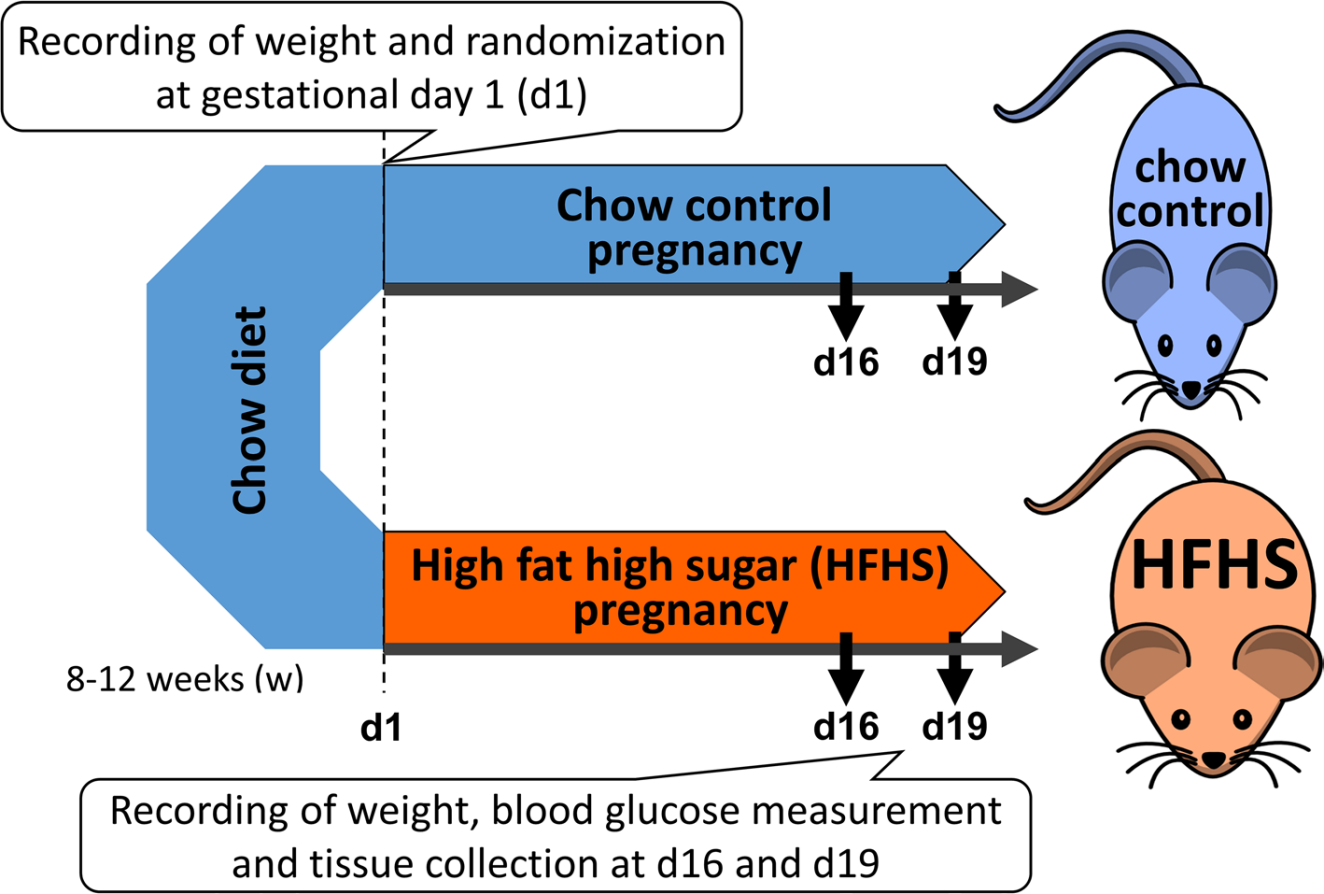
Schematic of the study design. C57Bl/6 J mice at 8–12 weeks age were mated with lean stud males. The day a copulatory plug was observed was defined as gestational day 1 (d1). On d1, females were randomly assigned to carry on either chow diet or fed the high fat high sugar (HFHS) diet until tissue collection at d16 or d19. Recording of maternal body weight was done at d1 and blood glucose measurement and tissue collection at the day of dissection

In brief, male and female C57BL/6 mice were housed under a 12:12 h dark/light photocycle with free access to water and the standard chow diet [chow: fat, 11%; protein, 26%; carbohydrate, 62% (simple sugar, 7%); 15.3 MJ/kg]. At 8–12 weeks of age, 42 females were mated with males and the day a copulatory plug was found was determined as d1 (Fig. 1). Pregnant females were singly housed from d1 onwards and assigned randomly to either continue feeding on the standard chow diet (n = 21) or fed a diet composed of processed ingredients high in saturated fats and simple sugars (HFHS diet: fat, 30%; protein, 17%; carbohydrate, 53% (simple sugar, 36%); 18.3 MJ/kg, n = 21) (Fig. 1). The details of the diets, namely nutritional composition and food intake have been reported previously [46]. According to the manufacturer, the diets contained 188 mg/kg and 49 mg/kg iron content, respectively. Based on the daily food intake of the dams [46], daily iron intake was 0.827 mg/day for the control chow and –0.162 mg/day for the HFHS diet-fed mice. On d16 or d19, dams were anaesthetized (intraperitoneal injection of 10ul/g fentanyl-fluanisone, midazolam [Janssen Animal Health, Belgium] in sterile water, 1:1:2), and then schedule 1 killed by cervical dislocation. Maternal organs (liver, kidneys, heart, and skeletal muscle [*biceps femoris*]) were collected, and conceptuses were dissected. All fetuses and placentas were weighed and collected. The placenta that was closest to the litter mean was bisected and both halves were fixed in 4% paraformaldehyde for histological assessment. The remaining placentas in the litter were snap frozen as a whole and stored at -80°C for further molecular and biochemical analyses.

### Iron content analysis by colorimetric ferrozine-based assay

Total iron content in liver and placental tissue lysates was determined using a colorimetric ferrozine-based assay [49]. Placental and maternal liver tissue lysates were prepared by homogenisation in RIPA lysis buffer as described previously [46]. Protein concentration in the lysates was determined using the Bicinchoninic Acid protein assay (Thermo Scientific, US). Then, 100 mM NaOH was added in equal volume to the samples, before iron releasing reagent (solution of equal volumes of 1.4 M HCl and 4.5% weight/volume (w/v) KMnO_4_, in distilled water) was added in two-times the volume of the samples. Samples were sealed with foil and incubated for 2 h at 60 °C. After cooling the samples to room temperature, iron detection reagent (6.5 mM ferrozine; 6.5 mM neocuproine; 2.5 M ammonium acetate; 1 M ascorbic acid; all dissolved in distilled water) was added in a 1:10 ratio (v/v). Samples were then incubated at room temperature for 30 min before absorbance was measured at 550 nm using a microplate reader (V_Max_ Kinetic ELISA Microplate Reader with Softmax Pro Software, Molecular Devices LLC, USA). Samples were run in duplicate and iron content interpolated using a FeCl_3_ standard curve. Maternal skeletal muscle iron context was determined using a QuantiChrom iron assay kit according to the manufacturer’s instructions (DIFE-250, BioAssay Systems, Hayward, CA 94545, USA). Iron contents were normalized to the protein concentration.

### Hepcidin measurement in maternal liver tissue lysate using enzyme-linked immunoassay (ELISA)

A mouse HAMP ELISA Kit (catalogue number: OKEH01408, Aviva Systems Biology, San Diego, CA 92121, USA) was used to quantify the levels of hepcidin in the maternal liver. Hepcidin quantification was performed according to the manufacturer’s recommendations and values were normalized to the protein concentration.

### Histological analysis of iron deposition by Prussian blue staining

Placental halves fixed in 4% paraformaldehyde were processed and embedded in paraffin wax using standard histological procedures [50]. Paraffin-embedded placentas were cut at 5 μm thickness, deparaffinized, rehydrated and stained with Prussian blue (1% potassium hexacyanoferrate and 1% HCl) followed by counterstaining with nuclear fast red. Slides were scanned using a Pannoramic Midi digital slide scanner (3DHISTECH Ltd.) and analysed under 10 × magnification using ImageJ (https://imagej.nih.gov/ij/). In brief, images of each placental cross-section were subdivided into decidua, junctional zone, and labyrinth zone, which correspond to maternal, endocrine and transport compartments of the placenta, respectively. Brightness of the blue regions, representing the histochemical detection of iron, was determined by automatically converting pixels in the threshold-based blue area to unweighted grayscale. The Macro-language of ImageJ was used to quantify the percentage of area occupied by iron in each section by applying “Colour Thresholder 1.53e” and the “Analyze Particles” functions. To measure both total tissue area (nuclear fast red positive) and iron deposition area (Prussian blue positive), the threshold limits were adjusted to select and quantify the blue iron-positive and the magenta area of the section as background. Finally, the iron deposition area in all three placental zones was normalized to the background within each section.

### Histochemical lipofuscin detection by Sudan Black B staining

Sudan-Black-B (SBB) stains lipofuscin, which consists of oxidized and cross-linked proteins, lipids, and metal ions, including iron that accumulate in the cytosolic compartment of cells [51]. After deparaffinization and rehydration, placental sections were stained with freshly prepared and filtered 60% Sudan Black B solution in ethanol (0.7% in 70% ethanol [w/v]) [51] before counterstaining with Nuclear Fast Red. Slides were scanned using a Hamamatsu NanoZoomer 2.0-HT slide scanner and analysed under 10 × magnification. Cells exhibiting dark navy-blue intracellular particles were considered lipofuscin-positive, and the percentage of SBB-positive tissue areas was determined by manual selection and subsequent quantification and normalization with background using the ImageJ macro (Threshold analysis).

### RNA isolation, reverse transcription, and quantitative PCR (qPCR)

Placental RNA was extracted using the RNeasy Plus Mini Kit (Qiagen, Manchester, UK) and reverse transcribed using Multiscribe Reverse Transcriptase with random primers (Applied Biosystems, Foster City, CA, USA). The cDNA was analysed in duplicate by qPCR using specific primers (Table 1), SYBR Green detection chemistry (GoTaq^®^qPCR Master Mix, Promega) on a ViiA7 thermocycler (Applied Biosystem) using default cycling conditions for 40 cycles (95 °C for 10 min, 95 °C for 15 s, 60 °C for 1 min). Melting curve analysis was used to verify the absence of additional PCR products and primer dimers. The expression of genes of interest were calculated using −ΔC_t_ by ensuring values were normalised to the mean expression of reference genes (*Ywhaz*, *Gapdh*, *Ubi*, and *Tbp*). These four genes remained stably expressed between the groups and represented relative to the control group mean.

**Table 1:** Primer list

Gene name	NCBI accession	Forward primer [5′–3′]	Reverse primer [5′–3′]	Product [bp]
Iron endocytosis gene				
*Trf1*	NM_011638.4	GGCGCTTCCTAGTACTCCCT	ACTTGCCGAGCAAGGCTAAA	119
Iron transporter genes				
* Dmt1*	NM_001146161.1	CAGGAGGTTGACTGGGTCG	GAATAGGATTCGGCTCCGCC	87
* Zip8*	NM_001135150.1	GGGACTAGCTTTCGGCATTT	GCATGTCGTTCATCTCTGGA	122
*Zip14*	NM_001135151.1	GGACCGCTATGGAAAGAATGAC	CTCACTCGCCCCGATCTG	196
*Fpn1*	NM_016917.2	TGCAGGAGTCATTGCTGCTAG	TGGAGTTCTGCACACCATTGAT	119
Oxido-reductase genes				
*Heph*	NM_010417.2	GAATTTTGCGAGCCGACCTT	TCATCCGCTTTCAGATACCC	111
*Cp*	NM_007752.3	TCTTGGAATCCTAGGTCCTGTC	TGAGGAGCGACCTGGTG	160
*Zp*	NM_001164797.1	CTACGACAAGGATTCGGAAGGAG	GGGCATCAATGTGTGAGTGG	182
Iron regulator genes				
*Hamp*	NM_032541.2	CAGGGCAGACATTGCGATAC	TGCAACAGATACCACACTGGG	113
*Hfe*	NM_010424.5	CAGCTGAGGACATGAGCCTA	GTATCTTAGAGAATGTGAACGCGG	119
Iron sensing, mRNA binding genes				
*Irp1*	NM_007386.2	AGCCTTTGGGAGTGAACGC	GATGACATGCTGCCTTTCCAC	98
*Irp2*	NM_022655.3	CCGGGGATCGTGTGATTCTG	ACACTGTCTCAGGTTCAGGC	122
Anti-ferroptotic genes				
*Cat*	NM_009804.2	AGACAATGTCACTCAGGTGCG	GCGTGTAGGTGTGAATTGCG	230
*Fsp1*	NM_012019.3	CGAGGAGTGATCGCCGAAAT	TGCTGGAACAAGTTGCCTGG	137
*Gclc*	NM_012815.2	AAGCCATAAACAAGCACCCC	CGGAGATGGTGTGTTCTTGTC	116
*Gss*	NM_012962.1	ATGCCGTGGTGCTACTGATT	TCTTCGGCGGATTACATGGA	107
* Gpx4*	NM_008162.4	GTGCATCCCGCGATGATTGGCG	GATTACTTCCTGGCTCCTGCCTC	255
* Hif1a*	NM_001313919.2	TTGGCAGCGATGACACAGAA	TGCAGGATCAGCACTACTTCG	167
*Pla2g6*	NM_001199023.1	GTGACCGCATTCTTCTCCGT	CCCCTCTGCTCTGGGTCA	377
Pro-ferroptotic gene				
*Acsl4*	NM_053623.1	CTCCTGCTTTACCTACGGCT	ACAATCACCCTTGCTTCCCT	97
*Nox2*	NM_007807.5	GACACGCATGCCTTTGAGTG	CGCCTATTGTGGTGTTAGGGT	262
*Xdh*	NM_011723.3	TCTATGCATCCAAGGCTGTCG	CTGGGGAGCCTCCTTTTCAG	258
Reference genes				
*Ywhaz*	NM_011740.3	TTGATCCCCAATGCTTCGC	CAGCAACCTCGGCCAAGTAA	88
*Gapdh*	NM_001289726.1	GAAGGGCTCATGACCACAGTC	CACGTCAGATCCACGACGG	227
* Ubi*	NM_019639.4	CCCACACAAAGCCCCTCAAT	AAGATCTGCATCGTCTCTCTCAC	70
*Tbp*	NM_013684.3	CCCTATCACTCCTGCCACAC	CTGCAGCAAATCGCTTGGG	158

### Measurement of lipid peroxidation products (TBARS)

The level of lipid peroxidation was measured in tissue lysates using a thiobarbituric acid reactive substances (TBARS) assay and expressed as concentration of lipid peroxidation equivalent product malondialdehyde (MDA) [52]. Briefly, the lysates were precipitated by mixing with 15% (w/v) trichloroacetic acid (TCA, Merck, Germany) in 1:1 (v/v) ratio. The precipitate was kept for protein carbonylation measurement (see below) and protein quantification (Pierce BCA Protein Assay, Thermo Fisher Scientific). The supernatant of the sample was mixed with thiobarbituric acid (TBA) solution (0.67% w/v TBA (VWR, Switzerland) in 2.5 M HCl) in a 1:1 (v/v) ratio, and boiled for 20 min at 95 °C. After cooling the samples to room temperature, 1-butanol was added with gentle mixing. Samples were then centrifuged at 1000 rcf and the butanol layer (top phase) was retrieved for measurement in a black-wall 96-well plate using a Flex Station II fluorescence reader (Thermo Fisher Scientific) at an excitation/emission wavelength of 530/550 nm, respectively. MDA equivalents were calculated by interpolation to an MDA (1,1,3,3 Tetraethoxypropane) standard curve. Concentrations of TBARS were expressed as MDA equivalents and normalized to protein concentration.

### Measurement of protein carbonylation

Protein carbonyl groups were measured by their reaction with 2,4-di-nitrophenyl-hydrazine (DNP) [53]. In brief, proteins in placental lysates (preparation see "Iron content analysis by colorimetric ferrozine-based assay" section) were isolated by TCA precipitation as described above for the TBARS assay. Subsequently, 10 mM DNP in 2 M HCl was added and samples were allowed to stand for 1 h at room temperature with intermittent vortexing. Thereafter, 20% (w/v) TCA was added for precipitation, followed by three washing steps with ethanol-ethyl-acetate (1:1) to remove free reagent. The precipitated proteins were dissolved in 600 µL of 6 M guanidine solution. The carbonyl content was spectroscopically determined and calculated from the optical density values at 405 nm measured by a Vmax Kinetic ELISA Microplate Reader (VWR) using a molar absorption coefficient of 22,000 M^−1^ cm^−1^ [53]. Carbonyl contents were normalized to the protein concentration (Pierce BCA Protein Assay, Thermo Fisher Scientific).

### Measurement of total glutathione (GSH) levels

To estimate the antioxidant potential of placental lysates, total glutathione levels were quantified as described previously [54]. GSH content was normalized to the lysate protein concentration.

### Assessment of proteins involved in stress signalling and ferroptosis activation by immunoblotting

Tissue lysates (preparation see"Iron content analysis by colorimetric ferrozine-based assay" section) were separated by SDS-PAGE and transferred onto 0.2 μm nitrocellulose membranes (Bio-Rad Laboratories, US) using a semi-dry technique (Semi-dry Blotter, Invitrogen) as described before [55]. Briefly, membranes were stained with Ponceau red, and staining captured on an iBRIGHT gel/membrane imager (Thermo Scientific, US). The membrane was washed with tris-buffered saline containing tween (TBST) and blocked with 5 % milk or fetal bovine serum (used for phosphorylated proteins) in TBST on a shaker, for 60 min at room temperature. Membranes were then incubated overnight at 4 °C using the following primary antibodies anti-Catalase (D5N7V) antibody (#14097, Cell Signaling Technology, Danver, MA, USA), anti-TFR1/CD71 (D7G9X) XP antibody (#13113, Cell Signaling Technology), anti-ERK1/2 antibody (#4695, Cell Signaling Technology), anti-phospho ERK1/2 antibody (#9911, Cell Signaling Technology), anti-p38 MAPK antibody (#8690, Cell Signaling Technology), anti-phospho-p38 MAPK antibody (#4511, Cell Signaling Technology), anti- JNK antibody (#9252, Cell Signaling Technology), anti-phospho-JNK antibody (#4668, Cell Signaling Technology). Thereafter, membranes were washed and incubated with rabbit or mouse secondary antibodies tagged to horseradish peroxidase (NA934 or NA931, Sigma Aldrich) diluted in TBST containing 2.5 % milk for 60 min. Following membrane washing, protein bands were visualised using Scientific SuperSignal West Femto enhanced chemiluminescence (ECL) substrate (Thermo Scientific, US) and captured on the iBRIGHT. The signal intensity of protein bands was quantified using ImageJ software and normalized to the Ponceau red signal, which is indicative of protein loading [55]. The abundance of phosphorylated proteins was calculated as a ratio to their respective total protein abundance.

### Statistical analysis

Statistical comparisons and the plotting of results were performed using GraphPad Prism (GraphPad) or SAS/STAT Software (Statistical System Institute Inc. Cary, NC, USA). Data are represented as column bars or Tukey boxplots. In the boxplots, the difference between the 25th and 75th percentiles are defined as inter-quartile range (IQR) and represented as a box, the median as line (-) and the average as plus sign ( +) in the IQR box. The limit of the upper whiskers marks the largest value if this value is smaller than the 75th percentile plus 1.5 times IQR and the lower whiskers the smallest value in the data set if bigger than the 25th percentile minus 1.5 IQR. Any values that fall beyond the whiskers of a box-and-whiskers plot are recognized as extreme values and plotted as individual points.

Statistical testing for normality was performed for each data set by D’Agostino-Pearson and Shapiro–Wilk normality test. For statistical analysis of maternal data, two-way ANOVA followed by Tukey’s multiple comparisons test was applied. Litter size was included as a covariate in the model. Feto-placental weights were analysed by ANOVA with repeated measures (MIXED model) with Tukey–Kramer's multiple comparisons test using the SAS/STAT Software. Each mother was considered the subject, the diet the main factor, and litter size was added as covariate. For the biochemical, histological and molecular analyses data were analysed by Mann–Whitney test to compare the HFHS to the control chow fed group at the respective study age. The ratios of placental and maternal liver iron content were analysed using an unpaired t-test. Per group and diet, one to two placentas per litter were analysed. A P value < 0.05 was considered as statistically significant.

## Results

### HFHS diet in pregnancy induces maternal obesity and affects placental and feto-placental weight

**Fig. 2:**
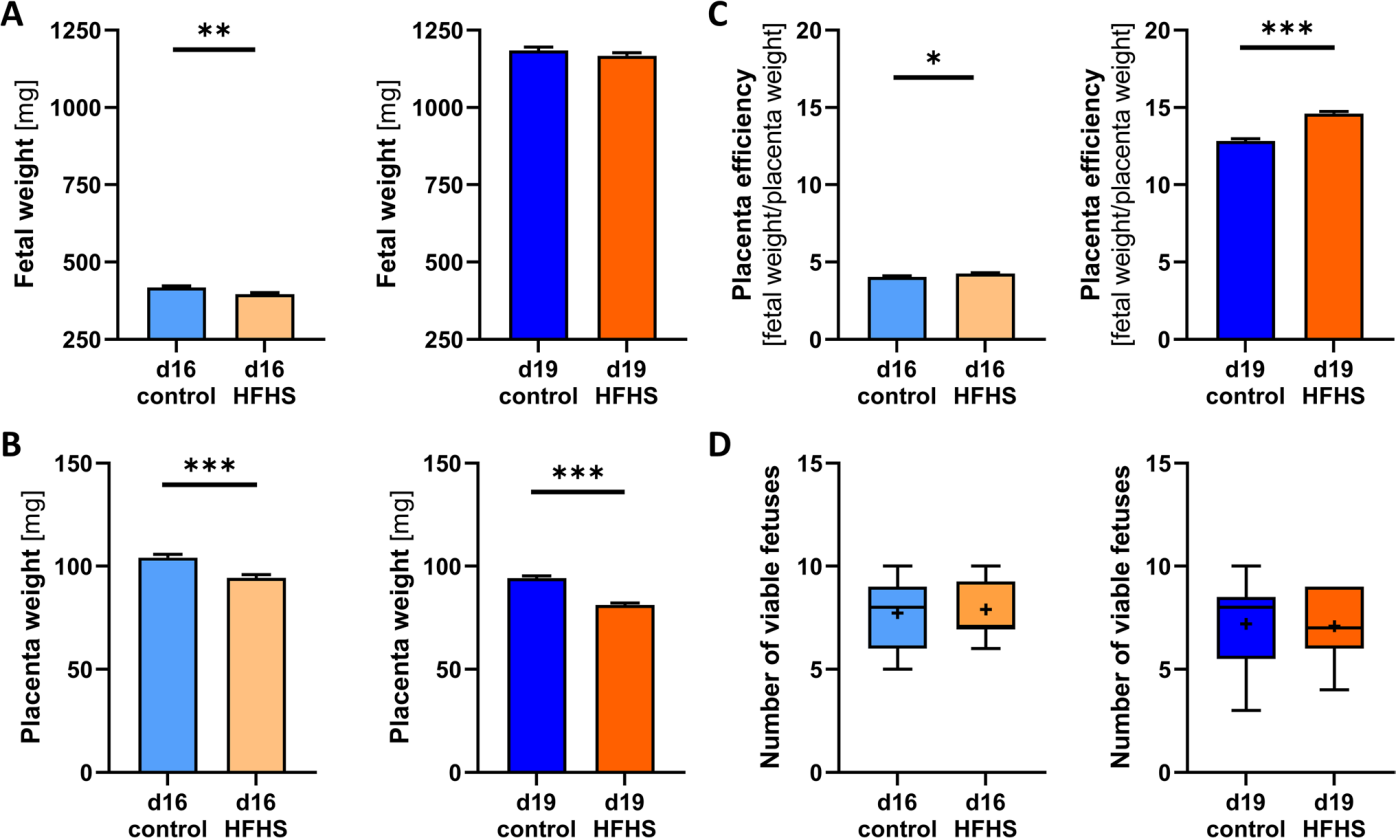
The effect of a HFHS diet on fetal and placental growth on day 16 and 19 of pregnancy. Fetal weights (**A**; n = 10–11/diet/age), placental weights (**B**; n = 10–11/diet/age), placental efficiency as calculated by the ratio between fetal and placental weight (**C**; n = 10–11/diet/age) were analysed by ANOVA with repeated measures (MIXED model) using the mother as the subject, the diet the main factor, and litter size as a covariate; α = 0.05, *P < 0.05, **P < 0.01, ***P < 0.001. Data are represented as estimated means and standard errors. The number of viable fetuses per litter (**D**; n = 10–11/diet/age) is represented as individual points, with points plotted beyond the whiskers of the box-and-whiskers plots displaying extreme values larger than the 75th percentile plus 1.5-times inter-quartile range or smaller than the 25th percentile minus 1.5-times inter-quartile range

The HFHS diet significantly reduced fetal weight on d16, whilst on d19 fetal weight was no longer affected (Fig. 2A). Placental weight was decreased on both d16 and d19 in the HFHS group (Fig. 2B). Placenta efficiency, defined as the ratio between fetal and placental weight was greater for the HFHS compared to control mice at both gestational ages (Fig. 2C). Litter size, defined as number of viable fetuses per litter, was not different among the groups at either gestational age (Fig. 2D). Hysterectomised bodyweight was reduced and retroperitoneal fat increased by HFHS diet during pregnancy (Table 2). Maternal liver and kidney weights were reduced, while heart weight did not show any significant changes in response to HFHS diet consumption (Table 2). Further, we found that the HFHS diet was associated with an increase in maternal circulating glucose concentrations (fed conditions) during pregnancy (Table 2). Thus, a HFHS diet induces elevated adiposity and hyperglycaemia in the mother during pregnancy and impacts fetal and placental growth in a gestational-age dependent manner.

**Table 2:** Effect of a HFHS diet on maternal organ weights at day 16 and 19 of pregnancy

	d16						d19						P^†^ value		
	Control			HFHS			Control			HFHS					
	Mean	± SE	n	Mean	± SE	n	Mean	± SE	n	Mean	± SE	n	Inter-action	GA	Diet
Body weight at conception [g]	18.55	± 0.56	6	18.55	± 0.53	7	19.27	± 0.62	5	19.46	± 0.62	5	0.8735	0.1784	0.8721
Body weight at study age [g]	30.80	± 0.65	11	28.99	± 0.68	10	37.43	± 0.68	10	35.07	± 0.65	11	0.6845	** < 0.0001**	**0.0033**
Body weight post hysterectomy (p.h.) [g]	23.61	± 0.64	11	22.40	± 0.67	10	25.33	± 0.67	10	23.16	± 0.64	11	0.4666	0.0675	**0.0135**
Blood glucose fed state [mM]	10.85	± 1.51	11	12.51	± 2.38	10	8.71	± 1.93	10	10.53	± 1.84	11	0.8876	**0.0017**	**0.0063**
Retroperitoneal fat weight [g]	0.08	± 0.01	9	0.11	± 0.01*	10	0.06	± 0.01	10	0.08	± 0.01	11	0.3308	**0.0131**	**0.0047**
Retroperitoneal fat (% body weight p.h.)	0.33	± 0.04	9	0.50	± 0.04*	10	0.26	± 0.04	10	0.35	± 0.04	11	0.4082	**0.0092**	**0.0023**
Liver weight [g]	1.90	± 0.06	11	1.72	± 0.06	10	1.91	± 0.06	10	1.67	± 0.06*	11	0.6229	0.8217	**0.0011**
Liver (% body weight p.h.)	8.02	± 0.28	11	7.67	± 0.30	10	7.66	± 0.29	10	7.24	± 0.28	11	0.9129	0.1799	0.1833
Kidney weight [g]	0.27	± 0.01	9	0.24	± 0.01	8	0.29	± 0.01	9	0.26	± 0.01	10	0.9743	**0.0458**	**0.0016**
Kidney (% body weight p.h.)	1.14	± 0.03	9	1.05	± 0.03	6	1.16	± 0.03	9	1.11	± 0.03	10	0.4019	0.1663	**0.0122**
Heart weight [g]	0.13	± 0.01	11	0.12	± 0.01	10	0.15	± 0.01	10	0.14	± 0.01	11	0.9940	**0.0104**	0.1504
Heart (% body weight p.h.)	0.55	± 0.02	11	0.54	± 0.02	10	0.58	± 0.02	10	0.59	± 0.02	11	0.4337	**0.0230**	0.9240

Data are represented as mean ± standard error (SE). †, P value of two-way ANOVA analysis with litter size as a covariate, α = 0.05; P  <0.05 are shown in bold font. Overall significant effects were followed by Tukey’s multiple comparisons, *P < 0.05 in ± SE columns

### Maternal HFHS diet does not change maternal iron status, but affects placental iron distribution and deposition in pregnancy

**Fig. 3:**
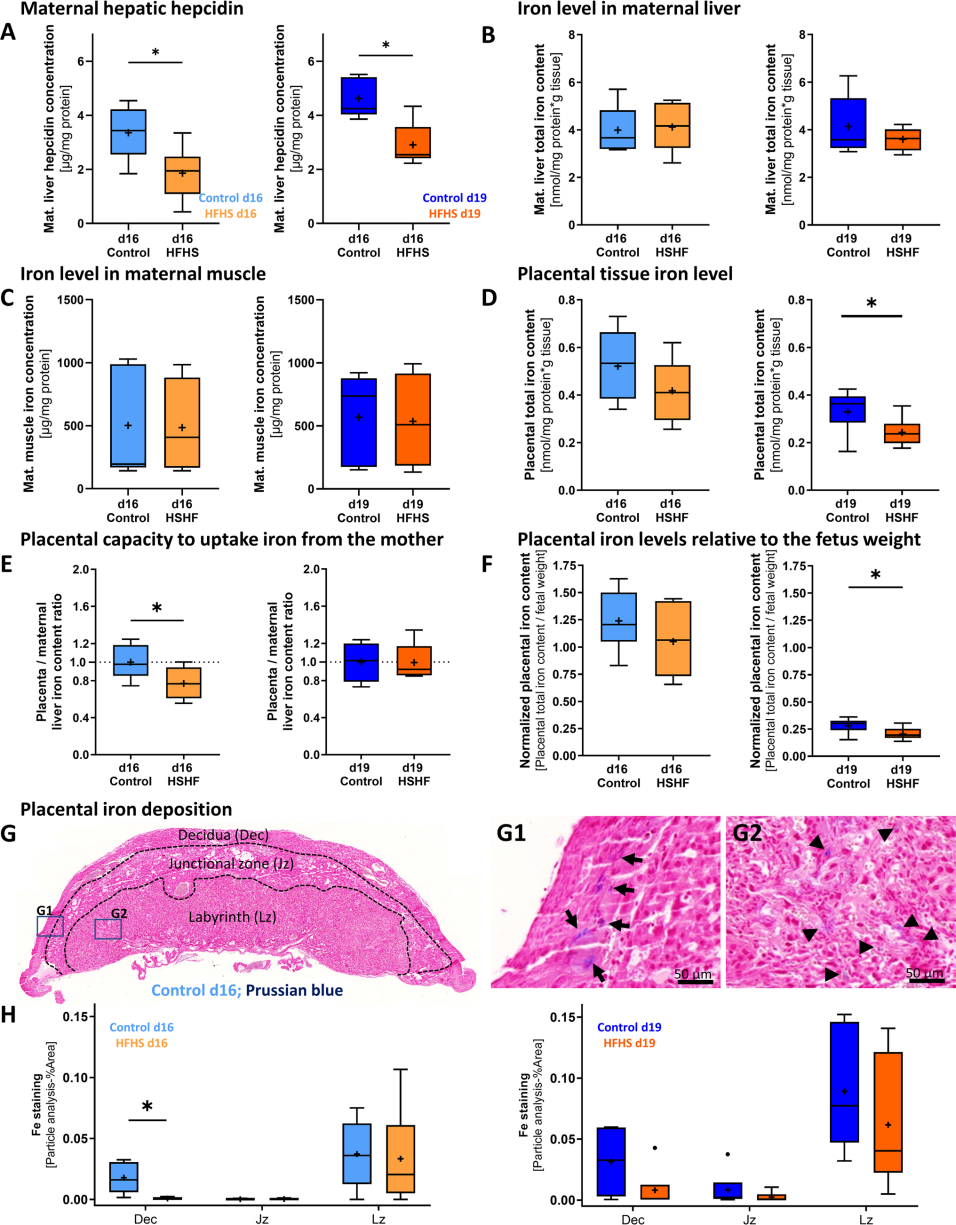
The effect of a HFHS diet on maternal hepatic hepcidin, maternal liver, skeletal muscle and placenta iron content and placental iron deposition on day 16 and 19 of pregnancy. The master iron regulator hepcidin was quantified by ELISA in maternal liver lysates (**A**; n = 5–6/diet/age). Tissue iron content was measured by ferrozine-based assay or QuantiChrom Iron kit in liver (**B**; n = 5–6/diet/age), skeletal muscle (**C**; n = 5–6/diet/age) and placenta (**D**; n = 10–11/diet/age). The ratio of placenta to maternal liver iron content (**E**; n = 4–7/diet/age) was analysed as a parameter for placental iron uptake capacity. The placental capacity to transfer iron to the fetus is represented by placental iron content normalized to fetal weight (**F**). Tissue iron deposition was visualized by Prussian blue staining in mouse placenta cross-sections (**G**, **H**) and analysed using Image **J** (**H**; d16 n = 4–6 and d19 n = 6/diet). Dec = decidua, Jz = junctional zone, Lz = labyrinth zone. Arrows in G1-2 highlight blue-stained iron deposits in the decidua (**G1**) and arrow heads (►) focal iron deposits in the placental labyrinth (**G2**) of a control chow placenta from d16. In all boxplots, controls are shown in blue, HFHS dams in orange. Data are from 1–2 placenta per litter. Data are represented as Tukey box and whiskers and analysed by Mann–Whitney test, α = 0.05, *P < 0.05 or by unpaired t test (**E**). The individual points that are plotted beyond the whiskers of the box-and-whiskers plots are extreme values larger than the 75th percentile plus 1.5-times inter-quartile range.

Maternal hepcidin protein concentration was reduced by a HFHS diet at both gestational ages (Fig. 3A). However, maternal liver and skeletal muscle iron concentration was not different between HFHS and control dams at either d16 or d19 (Fig. 3B, C). At d16, placental iron content was also not altered, but at d19, it was reduced in HFHS dams (Fig. 3D). Placental capacity to take up iron from the mother, represented by the ratio of placental iron to maternal liver iron concentration, was reduced at d16, but found to be no longer affected on d19 in HFHS dams (Fig. 3E). Placental capacity to transfer iron to the fetus for growth, as indicated by placental iron content relative to fetal weight, was similar between HFHS and control dams at d16, but lower in HFHS than control dams at d19 (Fig. 3F). Histological analysis by Prussian blue staining revealed intense and numerous iron deposits in the placental labyrinth zone, with fewer in the junctional zone and decidua independent of the gestational diet (Fig. 3G, H). However, focal iron deposition in the decidua was significantly lower in HFHS mice compared to control mice at d16, with no significant change observed at d19 (Fig. 3H). There were no differences in iron deposition in the junctional or labyrinth zones in response to a maternal HFHS diet, regardless of the gestational age (Fig. 3H).

### Maternal HFHS diet alters iron homeostasis gene expression in the placenta

**Fig. 4:**
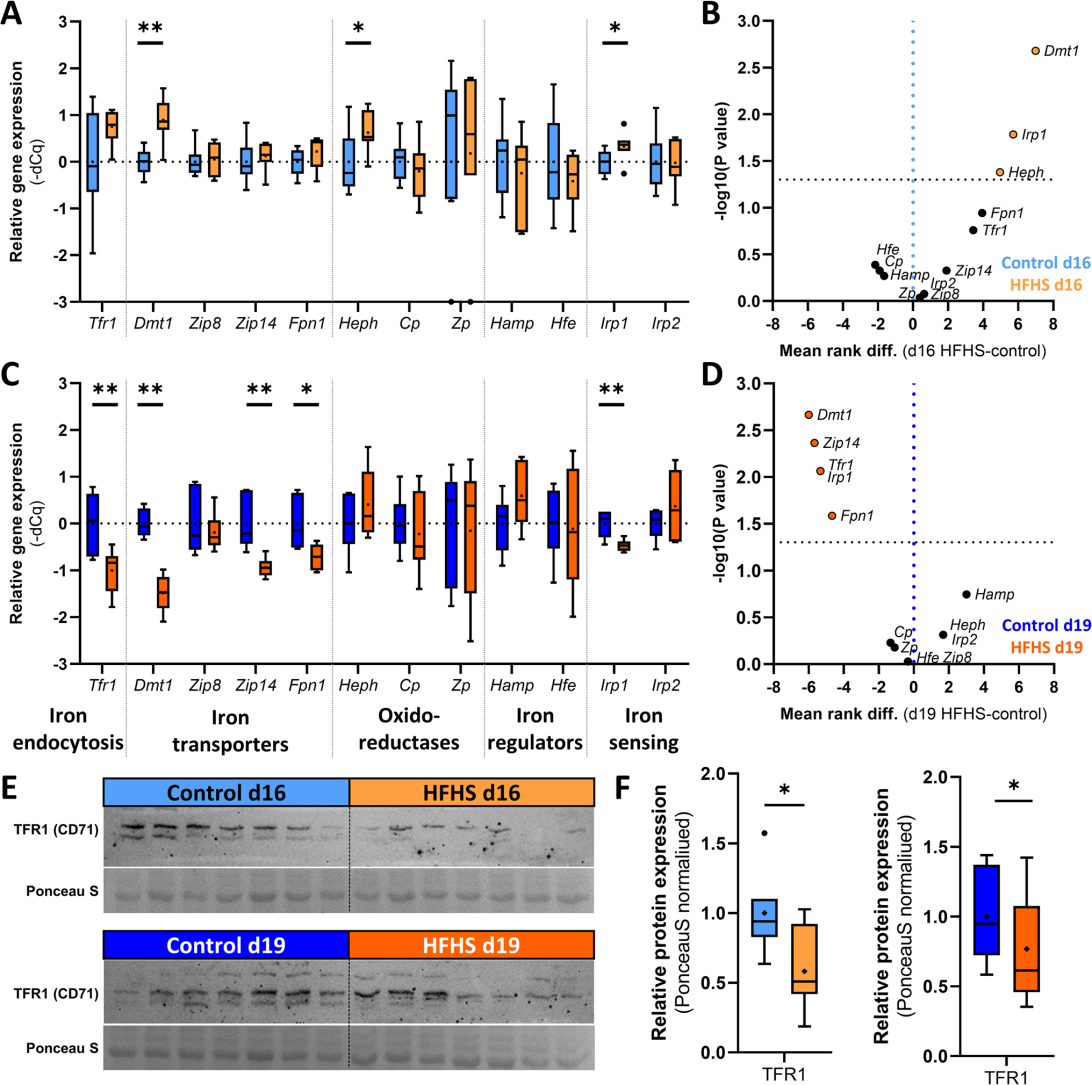
The effect of a HFHS diet on the placental expression of various iron homeostasis genes and proteins on day 16 and 19 of pregnancy. The mRNA levels of selected iron homeostasis genes were evaluated by RT-qPCR in placental tissues at d16 (**A**, **B**; n = 7–9/diet/age) and at d19 (**C**, **D**; n = 6/diet/age). Data are represented as Tukey box and whiskers (**A**, **C**) and analysed by Mann–Whitney test, α = 0.05. Additionally, gene expression levels are shown as volcano plots (**B**, **D**). The abundance of TFR1 (CD71) was determined by immunoblotting (**E**, **F**; n = 7/diet/age). In all charts, controls are shown in blue, HFHS in orange. Data are from 1–2 placenta per litter. The mean rank differences in the volcano plots were plotted on the x-axis, where negative values represent a down- and positive an up-regulation of mRNA levels in the HFHS relative to control placentas (blue dotted vertical line) and negative decade logarithm of the P-values are shown in y-axis, where the black horizontal dotted line represents α = 0.05 (− log_10_α = 1.301). *P < 0.05; **P < 0.01. The individual points that are plotted beyond the whiskers of the box-and-whiskers plots are extreme values larger than the 75th or smaller than the 25th percentile ± 1.5-times inter-quartile range

The analysis of 12 important iron homeostasis genes by RT-qPCR revealed an opposing effect of the HFHS diet on placental gene expression at d16 and d19 (Fig. 4A–D). There was a significant upregulation of the ferroxidase hephaestin (*Heph*), the iron-responsive regulatory protein (*Irp1)*, and the iron transporter divalent metal transporter 1 (*Dmt1*/*Slc11a2*) in the placenta of HFHS mice at d16 (Fig. 4A, B). In contrast, at d19 *Heph* was no longer significantly altered, iron transporters *Dmt1*, Zrt- and Irt-like protein 14 (*Zip14*) and ferroportin (*Fpn1*), as well as transferrin receptor (*Tfr1*) and *Irp1,* were downregulated in the placenta of HFHS dams (Fig. 4C, D). There was no effect of maternal HFHS diet on placental mRNA levels of iron homeostatic regulators hepcidin (*Hamp)* and *Hfe* at either gestational age. Informed by immunoblotting, protein levels of TFR1 (CD71) were reduced in the placenta at d16 and d19 in HFHS dams (Fig. 4E, F).

### Maternal HFHS diet results in dysregulation of ferroptosis and stress kinase signalling in the placenta

**Fig. 5:**
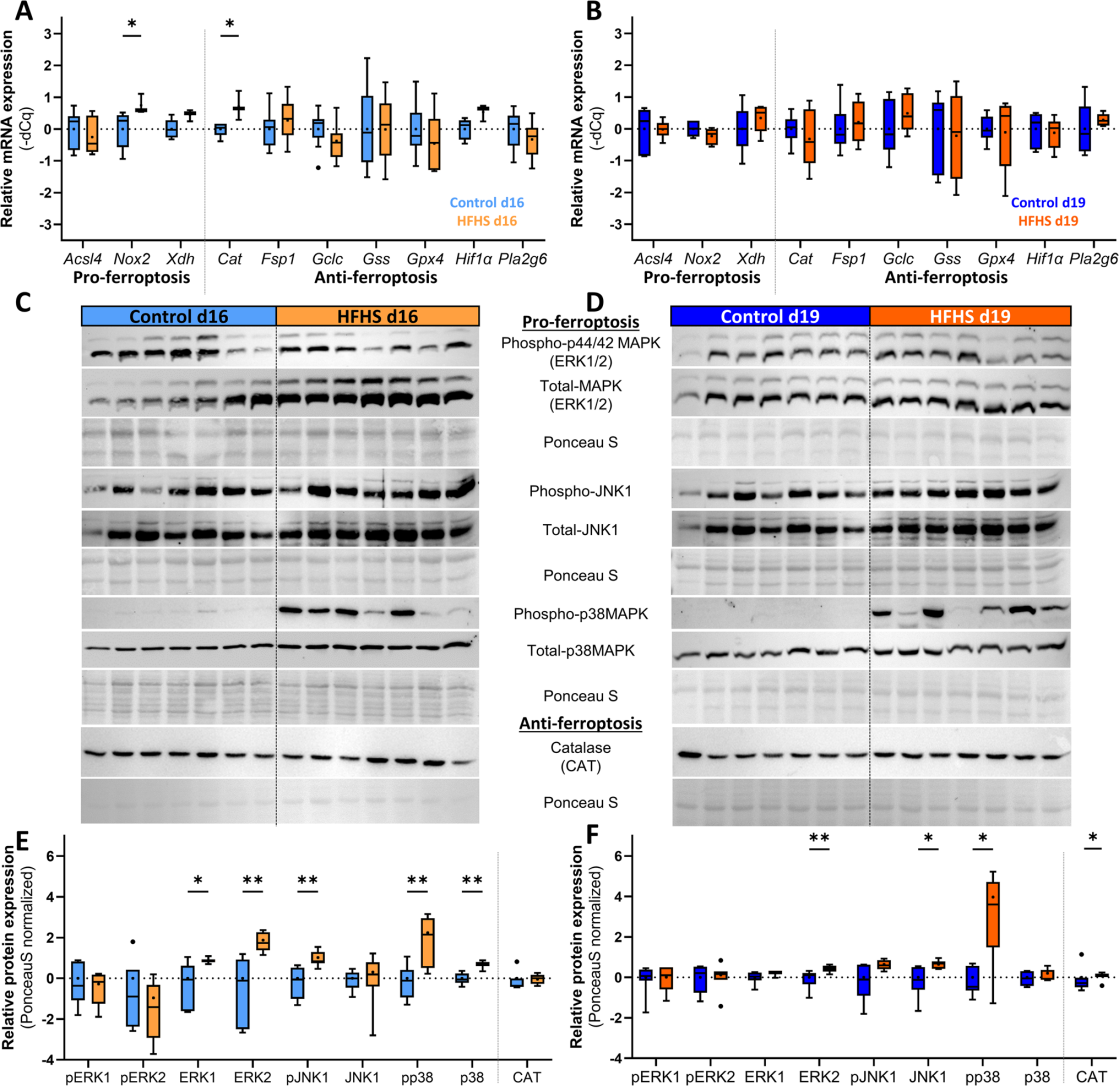
The effect of a HFHS diet on genes and proteins involved in ferroptosis in the placenta at days 16 and 19 of pregnancy. The expression of ferroptosis-specific genes was determined by RT-qPCR (**A**, **B**; n = 3–9/diet/age). Immunoblots with corresponding Ponceau S pictures are shown for d16 and d19 (**C**, **D**; n = 7/diet/age). The respective quantification of signalling proteins is displayed relative to the control group means (**E**, **F**). Data are from 1–2 placenta per litter. Data were analysed by Mann–Whitney test, with α = 0.05 and are represented as Tukey box and whiskers and normalized to the control (**C**–**F**). In all charts, controls are shown in blue, HFHS in orange. *P < 0.05; **P < 0.01. The individual points that are plotted beyond the whiskers of the box-and-whiskers plots are extreme values larger than the 75th or smaller than the 25th percentile ± 1.5-times inter-quartile range

As informed by RT-qPCR analysis, pro-ferroptosis and oxidative stress related gene NADPH oxidase 2 (*Nox2*) and anti-ferroptotic and antioxidant gene catalase (*Cat*), were significantly upregulated at d16 (Fig. 5A). The mRNA levels of the pro-ferroptosis marker xanthine dehydrogenase (*Xdh*) and hypoxia-inducible factor-1 alpha (*Hif1α*) which is a master regulator of responses to low oxygen, but can also regulate the expression of genes involved in the execution of ferroptosis [56] showed a non-significant tendency to be upregulated as well (Fig. 5A, both p = 0.071). These genes were no longer altered in the placenta at d19 by a maternal HFHS diet (Fig. 5B). Furthermore, the other pro-ferroptotic gene analysed, namely long-chain-fatty-acid-CoA ligase 4 (*Acsl4*), and other anti-ferroptotic genes assessed, in particular, ferroptosis suppressor protein 1 (*Fsp1*), glutamate-cysteine ligase (*Gclc*), glutathione synthetase (*Gss*), and calcium-independent phospholipase A2 beta (*Pla2g6*), were unaltered in the placenta at either d16 or d19 when comparing HFHS fed versus control placentas (Fig. 5A, B). However, ferroptosis is also mediated via changes in stress protein signalling pathways, namely the extracellular signal regulated kinase (ERK), c-Jun N-terminal kinase (JNK) and mitogen activated protein (MAP) kinase p38 families [57]. Hence, their abundance and activation levels in the placenta in response to a maternal HFHS diet were analysed by immunoblotting (Fig. 5C–F). This analysis revealed a significant upregulation of ERK2 and p38 proteins, in addition to elevated phosphorylated (activated) JNK1 and p38 in the placenta of HFHS mice at d16 (Fig. 5C–E) and at d19 (Fig. 5D–F). The upregulation of total ERK2 and phosphorylated p38 persisted until d19, while JNK1 total protein was increased in the placentas of HFHS mice at d19 only. The protein abundance of CAT although not different at d16, was found to be significantly upregulated in the placenta of HFHS mice at d19.

### Maternal HFHS diet reduces protein carbonylation but increases lipofuscin in the placenta at d16

**Fig. 6:**
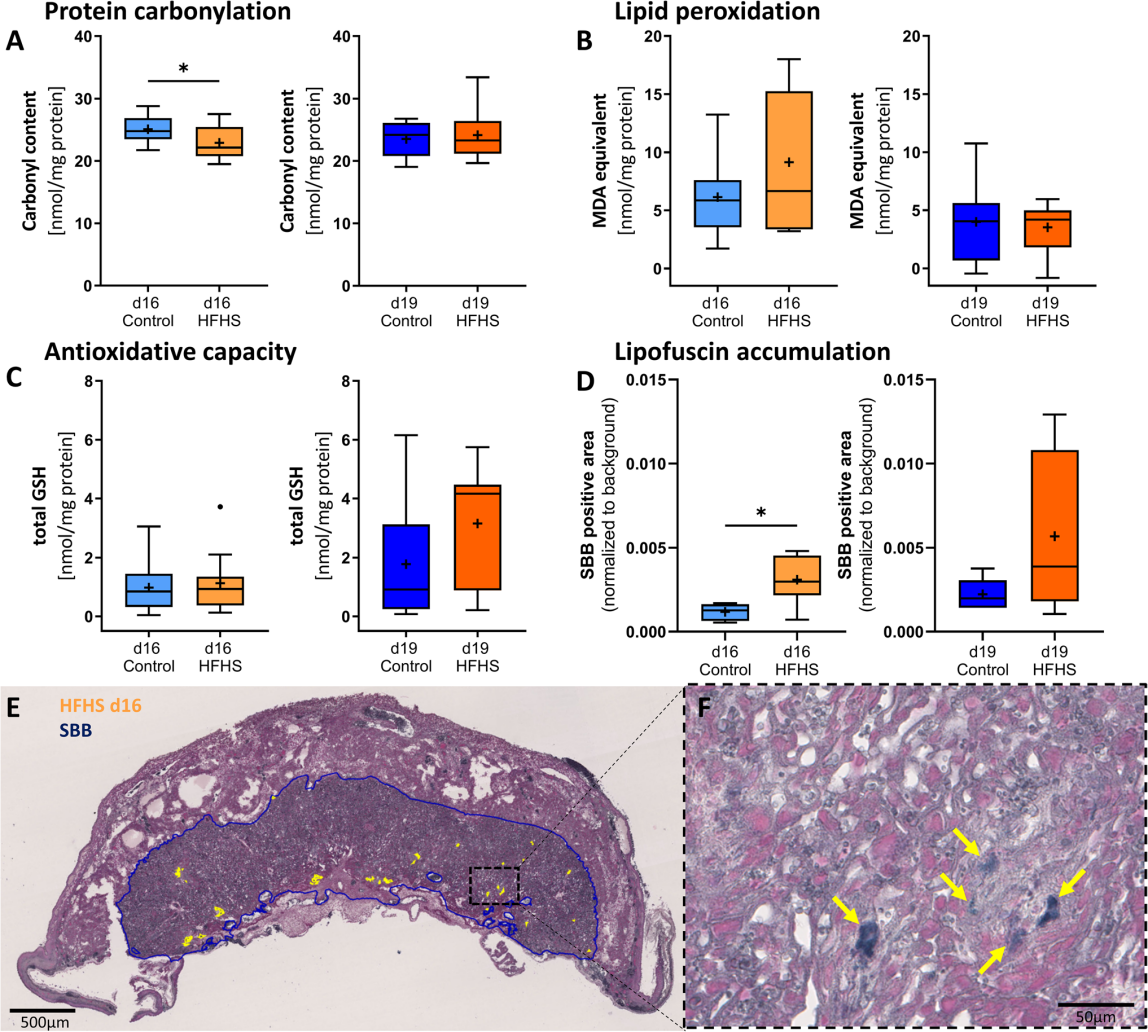
The effect of a HFHS diet on oxidative stress indicators and antioxidant capacity in the placenta at day 16 and d19 of pregnancy. Oxidative damage to lipids was measured by thiobarbituric acid reactive substances (TBARS) assay (**A**) and oxidative damage to proteins by 2,4-di-nitrophenyl-hydrazine (DNP)-mediated carbonyl quantification assays (**B**). The antioxidant potential was estimated by measuring the activity of total glutathione (GSH) (**C**). All assays were performed on placental tissue lysates from d16 and d19 (**A**–**C**; n = 10–11/diet/age). Furthermore, lipofuscin an indicator of iron-dependent oxidative damage and ferroptosis was assessed by histologic detection and quantification of Sudan-Black-**B** (**D**–**F**; n = 6–8/diet/age). A representative image of a HFHS placenta section at d16 is shown with the labyrinth depicted in blue and SBB-positive areas highlighted in yellow. Panel F demonstrates a high magnification (40x) close-up of the placental labyrinth, with yellow arrows pointing to SBB-positive lipofuscin. In all charts, controls are shown in blue, HFHS in orange. Data are from 1–2 placenta per litter. Statistical significance was determined using Mann–Whitney test, α = 0.05, *P < 0.05. The individual points that are plotted beyond the whiskers of the box-and-whiskers plots are extreme values larger than the 75th percentile plus 1.5-times inter-quartile range

There was no significant increase in lipid peroxidation, a marker of oxidative damage at either gestational age (Fig. 6A). In addition, protein carbonylation, also a marker of oxidative stress was significantly lower in the placenta of HFHS compared to control mice specifically at d16 (Fig. 6A, B). The activity of the antioxidant GSH in the placenta was also similar between HFHS and control mice at both d16 and d19 (Fig. 6C). However, lipofuscin formation, which is associated with iron-dependent oxidative damage and ferroptosis [58], was increased in the placental labyrinth at both gestational stages by a maternal HFHS diet, with the effect statistically significant at d16 (Fig. 6D, E).

## Discussion

The present study demonstrates that the maternal consumption of a HFHS diet during pregnancy, which causes elevated maternal adiposity and hyperglycaemia, alters placental iron homeostasis in association with changes in fetal growth. Furthermore, these changes were independent of a change in maternal iron status. In particular, the HFHS diet impacted placental iron transfer capacity, decreased placental iron content, and led to a dysregulation of genes involved in ferroptosis, increased iron-dependent oxidative damage and stress kinase activation in the placenta. Changes were dependant on gestational age and were related to reduced, and then subsequent normalised fetal growth in the HFHS diet-fed mice. Our results are largely in line with previous studies of the term, delivered human placenta from women who developed GDM [45], as well as studies exposing trophoblast cell lines to hyperglycaemic conditions in vitro [45]. Our findings, highlight the utility of this HFHS mouse model to study impacts of diet-induced maternal metabolic disease during pregnancy, as well as to inform on changes in placental iron handling with respect to fetal growth outcomes in vivo.

In the current study we found that fetal growth was reduced at d16 but was then unchanged towards term (d19) in HFHS fed mice. This is largely consistent with the altered pattern of fetal growth trajectory previously analysed in HFHS mouse cohorts [46, 47]. Moreover, like previous work using the HFHS mouse model, placental weight was reduced in the HFHS-fed mice [46, 59–61]. In our model, we have previously shown that glycogen storage by the placenta was reduced by  25 % in HFHS-fed mice [46], which is partly due to the depletion of glycogen stores in the placenta [62]. The recovery in growth of the fetus in late gestation despite a lighter placental weight in HFHS pregnant mice is most likely a result of metabolic adaptation of the placenta through up-regulation of materno-fetal glucose, fatty acid, and amino acid transport downstream of key signalling pathways, such as those involving insulin and the mechanistic target of rapamycin (mTOR) [46, 63–65]. Indeed, placental efficiency was found to be significantly enhanced in the HFHS fed mice at both d16 and d19 of pregnancy. Consistent with the relatively low content of iron in diets of industrialized countries [2, 5], our customized HFHS diet had a lower iron content than the control chow diet. Low dietary iron is associated with changes in fetal and placental size in numerous species, including rodents and humans [66]. Although, the estimated daily iron intake was around 5-times less in the HFHS group (chow control: 188 mg/kg food iron content × 4.4 g/day food intake = 0.827 mg/day iron intake per day; HFHS: 49 mg/kg food iron content × 3.3 g/day food intake = 0.162 mg/day iron intake per day), our data demonstrated that maternal tissue iron levels (in liver and skeletal muscle) were maintained when compared to the chow control group. This maintained maternal iron status was likely secondary to enhanced dietary iron absorption, through the hepatic down-regulation of the master iron regulator hepcidin in the HFHS-fed dams [67]. Whether this may relate to reductions in the weight of the maternal liver and kidney, key organs involved in iron homeostasis in mice fed HFHS diet is currently unclear. Furthermore, despite our findings, we cannot fully exclude the possibility that differences in dietary iron availability may have contributed to the changes in gestational outcomes seen in mice fed the HFHS diet.

The estimated placental capacity to uptake iron from the mother, as informed by the placental to maternal liver iron content ratio and reduced placental TFR1 protein expression, was reduced at d16 in HFHS mice. Since the fetus depends on iron for growth [66], the reduced placental iron uptake ability in HFHS fed mice likely contributed to the decrement in fetal weight at d16. It may have also contributed to the diminished placental weight towards term in the HFHS mice reported herein. At d19, placental TFR1 protein abundance was still reduced, but placental capacity to take up iron from the mother was no longer decreased. Placental iron content and placental iron content relative to fetal weight were both reduced, but fetal weight was enhanced in the HFHS group at d19. Previous work has reported that maternal consumption of this HFHS diet led to morphological changes, including reduced fetal capillary volume and increased interhaemal membrane thickness at d16 that were partially recovered by d19 [46]. Whether morphological changes explain the altered placenta iron handling and fetal growth trajectory in HFHS fed mice, however, will require further studies. Indeed, other work has suggested relationships between materno-fetal substrate exchange, placental morphometric changes and fetal growth outcomes in pregnancies associated with GDM [68], type-2 diabetes [69] and obesity [70]. In the present study, reduced placental iron acquisition was related to decreased iron deposition in the decidua of the placenta in HFHS diet-fed mice at d16. These data contrast previous findings where examination of gestational tissues from women with GDM revealed increased iron storage within the chorion and decidua, namely in macrophages that showed an M2-like profile [71]. Thus, further work is needed to assess the significance and cellular localisation of iron deposits in the placenta of the HFHS diet-fed mice. Moreover, it needs to be elucidated whether iron accumulates in the decidua due to reduced transport across the placenta in vivo [72], and/or whether changes could involve the low maternal hepcidin levels leading to greater release of cellular iron via FPN1 [67]. However, it is important to note that iron transport across the placenta and regulation of fetal iron levels are complex processes [16], so trying to identify precise changes and cause and effect relationships are likely to be challenging.

Altered placental iron handling was related to the disrupted expression of key iron homeostasis genes in the placenta of mice fed the HFHS diet. In this context, increased placental expression of *Dmt1*, *Heph* and *Irp1* at d16 may reflect an attempt to compensate for the impaired maternal iron uptake capacity of the placenta in HFHS mice at d16. For instance, in response to low cellular iron levels, IRP1 binds to the 3′-iron responsive element (IRE) stem-loop structure within untranslated regions of genes, that in turn, promotes the stabilization of mRNAs which increase iron uptake, namely *Tfr1* for iron endocytosis and *Dmt1* for the transport of ferrous iron across the endosomal membrane into the cytosol [73]. Furthermore, HEPH is a multi-copper oxidase with ferroxidase activity that is crucial for cellular iron release by the iron exporter FPN1 [74], as *Fpn1* is targeted for degradation in the absence of ferroxidase activity [75]. In contrast to the up-regulation of placental iron transfer genes at d16, there was a strong and harmonized down-regulation of the *Dmt1*, *Zip14*, *Fpn1*, *Tfr1* and *Irp1* at d19 in HFHS diet-fed mice. The opposing regulation of iron handling genes at the two gestational time points suggest a transition from increased to restricted materno-fetal iron transport between d16 and d19. However, the down-regulation of TFR1 protein persisted in the placenta of HFHS mice, and was seen at both d16 and d19. According to a recent gene cloning and ontology study, the TFR1 protein levels are predominately determined by the amount of *Tfr1* mRNA available for translation [76]. However, other regulating factors, such as post-transcriptional regulation through the interaction of IRPs as mentioned before is also possible [73]. In this case, the downregulation of *Irp1* and hence stabilization of *Tfr1* mRNA at d19 could explain the reduced TFR1 protein levels. Previous in vitro studies trying to mimic hyperglycaemic conditions of GDM in the human trophoblast cell line BeWo have shown an initial upregulation of *DMT1* and Zrt- and Irt-like protein 8 (*ZIP8*/*SLC39A8*) after 3 days, followed by a stable downregulation of *DMT1*, *FPN1* and *TFR1* mRNA after 20 days of continued hyperglycaemia exposure [45]. These results suggest that trophoblasts react upon stimulation with diabetic-like conditions by an initial upregulation, followed by a reduction of placental iron transfer mechanisms, that are consistent with our opposing observations between d16 and d19 in the placenta of HFHS mice in the present study. We have previously shown that iron homeostasis genes are altered by GDM in the term human placenta through an orchestrated downregulation of *DMT1* and *FPN1* mRNA, but also ZIP8 and TFR1 protein [45]. Interestingly, these changes were mostly reflected in murine placentas at term (d19) in response to the HFHS diet in the present study, underlining a conserved impact of disturbed glucose handling on placental iron homeostasis.

The lower iron content of the HFHS compared to chow control diet may have also contributed to the changes in iron homeostasis gene expression seen in the placenta. For instance, it has been previously shown that dams fed with iron deficient diet exhibit increased placental *Tfr1*, *Dmt1* and *Fpn1* expression [72, 77]. Although, obesity is associated with higher hepcidin and therefore lower dietary iron absorption [21], maternal iron status as measured by liver and muscle iron levels was not reduced in the HFHS diet-fed mice in the present study. Taken collectively, these findings indicate that the reduced placental iron uptake and tissue content and deposition in the present study resulted from changes in placental iron regulation, rather than from lower dietary iron levels or reduced iron absorption. As mentioned before, the reduced food iron availability in the HFHS diet was tackled by downregulation of hepcidin and likely increased dietary iron absorption. The observed alterations in placental iron handling genes may also reflect part of a protective mechanism that serves to prevent oxidative damage in the fetus and placenta. Indeed, lipid peroxidation in the placenta was not increased, and protein carbonyl formation was reduced at d16 in mice fed the HFHS diet. Despite this, lipofuscin, an alternative marker of oxidative stress was elevated in the placenta of HFHS-fed mice (significant effect seen at d16). Lipofuscin is known to accumulate iron, which in turn, can generate oxidative stress through the Fenton reaction and is associated with ferroptosis [58, 78]. As we found lower placental iron yet decreased protein carbonylation and increased lipofuscin, further work is required to understand the relationships between these factors in the placenta in the context of maternal obesity. Recent work using CRISPR-based functional genomics has found the lysosomal protein prosaposin (PSAP) is responsible for controlling survival during oxidative stress in neurons derived from induced pluripotent stem cells [78]. Notably, this study demonstrated that knockdown of PSAP resulted in the formation of lipofuscin, which traps iron, generates ROS and triggers ferroptosis [78]. Although PSAP was not measured in the current study, collectively these data suggest a similar mechanism may be in operation and could explain the disparities between iron mishandling and oxidative stress markers in the placentas from HFHS mice.

In general, oxidative damage occurs when ROS levels exceed antioxidant defences [3]. However, GSH, as the main antioxidative opponent of ferroptosis, was not significantly induced in dams fed the HFHS diet, neither through upregulation of *Gpx4* mRNA expression nor increased GSH activity. Interestingly, we found increased expression of the oxidative stress and pro-ferroptotic gene *Nox2* alongside increased expression or abundance of the anti-oxidant catalase in the placenta of HFHS dams. Our findings agree with others showing increased ROS levels and anti-oxidant imbalance in the placenta involving increased catalase expression in various rodent models where type-1 and type-2 diabetes were induced via genetic, surgical and pharmacological methods [79–81]. However, it is also likely that the present HFHS diet, which was just fed to the mother during pregnancy, was not sufficiently severe to provoke an overall increase in lipid peroxidation or to exhaust the antioxidative potential of the placenta. The present study demonstrates that maternal adiposity and/or elevated circulating glucose seems to induce mild oxidative damage in the placental labyrinth whilst simultaneously increases antioxidative potential via CAT up-regulation in late gestation. These findings also highlight that the length of HFHS diet feeding, magnitude of diet-induced obesity and/or hyperglycaemia exposure are important for determining placental oxidative responses.

Alongside with the detection of mild oxidative damage and an antioxidant imbalance in the placentas of HFHS diet-fed pregnant mice, signalling pathways, namely the MAPK pathway can act as indicators of ferroptosis activation in the placenta and trophoblast [16, 57]. The p38 and JNK1 branches of the MAPK family are particularly suspected to mediate ferroptosis downstream of lipid-related ROS [82], but this can be cell-specific [83, 84]. In the current study, the increased levels and/or activation of MAPK p38, JNK1 and ERK2 in the HFHS group may therefore indicate a degree of ferroptosis in the placenta [57]. We also found upregulation of *Nox2* by the placenta in HFHS mice, and NOX2 is implicated in the generation of ROS, is associated with ferroptosis [85] and is positively regulated by p38MAPK [86]. Prior work in a rat model of metabolic disease (insulin resistance) has also reported changes in iron deposition, activated ERK/p38/JNK and ferroptosis induction at the feto-maternal interface [87]. However, unlike that rat model of metabolic disease, which showed significantly augmented oxidative stress [87], the mRNA expression of anti-ferroptosis genes, including *Gclc*, *Gss* and *Gpx4 *[88] and other pro-ferroptosis genes, such as *Acsl4 *[89], were not significantly altered in the placentas from HFHS diet-fed mice at either gestational age. Therefore, it is possible that gestational changes in iron uptake by the placenta of HFHS diet-fed mice prevented a complete oxidative derailment, and in turn, a significant induction of ferroptosis. It is also important to note that in addition to their involvement in ferroptosis, MAPKs are involved in several other cellular processes. ERK kinases are responsible for trophoblast growth and differentiation, p38-kinases serve as mediators of oxidative stress pathways and apoptosis, and JNK, regulates apoptosis and immune cell responses [90]. Hence, further mechanistic studies are needed to understand the significance of the genes and pathways affected in the placenta and how these precisely relate to iron handling, ferroptosis and oxidative stress in the HFHS mice.

This integrative study of maternal and placental iron handling has key strengths that include examining changes at multiple levels (genes, proteins, enzyme activity, histology) at two key different stages of gestational development in an established diet-induced mouse model of metabolic disease during pregnancy. However, this study was limited by the lack of fetal tissues other than placenta, such as fetal haematopoietic organs and fetal blood. Therefore, knowing if fetal iron supply and status are affected in our model remains to be determined. Further work is also needed to causally test the association between fetal growth deviations and placental iron mis-handling. According to the National Research Council, 35 mg/kg dietary iron is needed to meet the requirements of nonpregnant mice [91]. However, higher concentrations may be necessary for pregnant animals, but also iron overload can be harmful [92]. Although the specific dietary iron requirement of pregnant mice is unclear, the daily intake of dietary iron was  0.83 mg/day for the control chow group (188 mg iron per kg chow diet) and  0.16 mg/day for the HFHS-fed mice (49 mg iron per kg HFHS diet), which could be considered iron-overload and iron-deficiency [72], respectively. As measured, maternal liver and muscle iron levels did not differ with the gestational diets. Future work is therefore required to fully understand the contribution of varied dietary iron intake and characterise maternal iron status in our gestational HFHS mouse model. Further studies should also consider strategies to exacerbate the maternal metabolic state, as well as the oxidative challenge of pregnancy, e.g., through additional glucose or iron to provoke a more pronounced pathological condition and/or by introducing the HFHS diet prior to pregnancy. However, identifying which particular physiological factors are responsible for the resultant effects (increased adiposity, inflammatory state of the placenta, metabolic derangements, altered placental substrate supply, increased placental oxidative stress, etc.) on iron homeostasis during pregnancy will be challenging [93, 94]. Alternatively, an induced ferroptosis model may be employed to study consequences for pregnancy outcomes, such as involving an engineered cyst(e)inase that causes systemic ferroptosis by degradation of both cystine and cysteine [95, 96]. Since we were not able to include groups in which different maternal parameters, such as maternal adiposity, hyperinsulinemia, hyperglycaemia, and dietary iron deficiency were solely modified, we do not know which of these variables, in isolation or in combination contribute to the outcomes we found in our HFHS mice. Therefore, much further work is required to identify the specific mechanisms underlying the changes in placental and fetal growth phenotype in response to maternal diet-induced obesity. Finally, fetal sex was not determined and therefore, the role of fetal sex in our outcome measures could not be assessed. This is a major limitation of the study and therefore will need to be addressed in future work, as fetal sex can influence the placental response to environmental/gestational insults [55, 94, 97–100]. This is particularly relevant given the emerging data showing there are differences in the phenotype of placenta of males and females from mice fed obesogenic diets from prior to pregnancy, and in women who have elevated adiposity during pregnancy [94, 101, 102].

### Conclusions

Collectively, our data show that gestational exposure to a HFHS diet alters placental iron homeostasis in association with changes in genes and proteins implicated in iron handling, stress and ferroptosis signalling, as well as feto-placental growth. These findings have implications given the increased intake of diets containing high amounts of fat and sugar in women at reproductive age in Western and industrialised societies and/or those women who change their food preference towards obesogenic diets during pregnancy. They may also have relevance more broadly for understanding possible consequences of the use, or lack, of iron supplementation in pregnant women. Most pertinently, these results are highly relevant given that the rates of obesity and GDM are increasing in many parts of the world and are associated with negative pregnancy outcomes and long-term effects on mother and child.

## Data Availability

The experimental data that support the findings of this study are available in Zenodo with the identifier https://doi.org/10.5281/zenodo.10202231.
